# Rare cytogenetic abnormalities in acute myeloid leukemia transformed from Fanconi anemia – a case report

**DOI:** 10.1186/1756-0500-6-316

**Published:** 2013-08-12

**Authors:** Shabneez Hussain, Salman Naseem Adil

**Affiliations:** 1 Section of Haematology, Department of Pathology and Microbiology, The Aga Khan University Hospital, Stadium Road, P.O. Box 3500, Karachi 74800, Pakistan

**Keywords:** Fanconi anemia, Acute myeloid leukemia, Cytogenetic abnormalities

## Abstract

**Background:**

Fanconi’s anemia (FA) is an inherited bone marrow failure syndrome that carries a higher risk of transformation to acute myeloid leukemia (AML) when compared with general population. AML is the initial presentation in approximately one third of patients.

**Case presentation:**

A 17 year old male presented to the emergency room with history of high grade fever since two weeks. Examination revealed pallor, short stature and thumb polydactyly. There was no visceromegaly or lymphadenopathy. Complete blood count showed haemoglobin 3.4 gm/dl, MCV 100 fl and MCH 36 pg, white blood cell count 55.9 × 10 E9/L and platelet count 8 × 10E9/L. Peripheral blood smear revealed 26% blast cells. Bone marrow was hypercellular exhibiting infiltration with 21% blast cells. Auer rods were seen in few blast cells. These findings were consistent with acute myelomonocytic leukemia. These blasts cells expressed CD33, CD13, HLA-DR, CD117, CD34 antigens and cytoplasmic myeloperoxidase on immunophenotyping. Bone marrow cytogenetics revealed 46, XY, t (8:21) (q22; q22) [11] / 46, XY, add (2) (q37), t (8; 21) [4] / 46, XY [5]. Molecular studies showed positivity of FLT 3 D835 variant and negativity of NPM 1 and FLT3 ITD (internal tandem domain) mutation. Peripheral blood analysis for chromosomal breakage exhibited tri-radial and complex figures. He received induction chemotherapy with cytarabine and daunorubicin (3 + 7). Day 14 marrow revealed clearance of blast cells.

**Conclusion:**

The recognition of specific cytogenetic abnormalities present in FA known to predispose to AML is crucial for early haematopoietic stem cell transplant (HSCT) before transformation to leukemia.

## Background

Fanconi anemia (FA) is an autosomal recessive disease which is the most frequent cause of inherited bone marrow failure syndromes. These patients usually present in the first and second decade of life [1] with bone marrow failure and various congenital abnormalities such as short stature, skin hyperpigmentation such as “cafe´-au-lait spots”, Fanconi facies, microphthalmia, cardiac, renal, genitourinary defects, thumb and radius deformities [2]. The initial change at birth in peripheral blood is macrocytosis without cytopenia. This is followed by thrombocytopenia and neutropenia. The incidence and prevalence of FA is not known in Pakistan. Studies from the West reveals that the incidence of FA is three per million [3]. The only case that has been reported form our region has been of FA transformation into acute lymphoblastic leukemia (ALL) [4].

Due to the increase in chromosomal instability, P53 activation and cell death; there is an increased possibility of clonal evolution in FA patients [5]. These patients present with malignancies at a younger age compared to that of general population and the predominant malignancies are AML, liver tumors, head and neck carcinomas and gynecological cancers [6]. The highest risk of myelodysplastic syndrome (MDS) and AML is in young adulthood [2,7] and they are usually preceded by an aplastic or hypoplastic phase.

The leukemia in FA differs from that of general population. In one retrospective study [8], it was found that approximately 27% patients presented with AML without being diagnosed with FA. The median age for developing leukemia was 14 years and the cumulative incidence of developing leukemia by the age of 29 years was 37%. Majority of the leukemias were AML (94%), predominantly acute myelomonocytic leukemia while remaining were ALL. This is in contrast with 84% of the leukemia being lymphoid in non-FA patients of similar age group.

We report a case of young male who presented with AML and based on his physical abnormalities, peripheral blood was analyzed for chromosomal breaks. He had rare cytogenetic abnormalities not seen in AML transformed from FA.

## Case presentation

**Figure 1:**
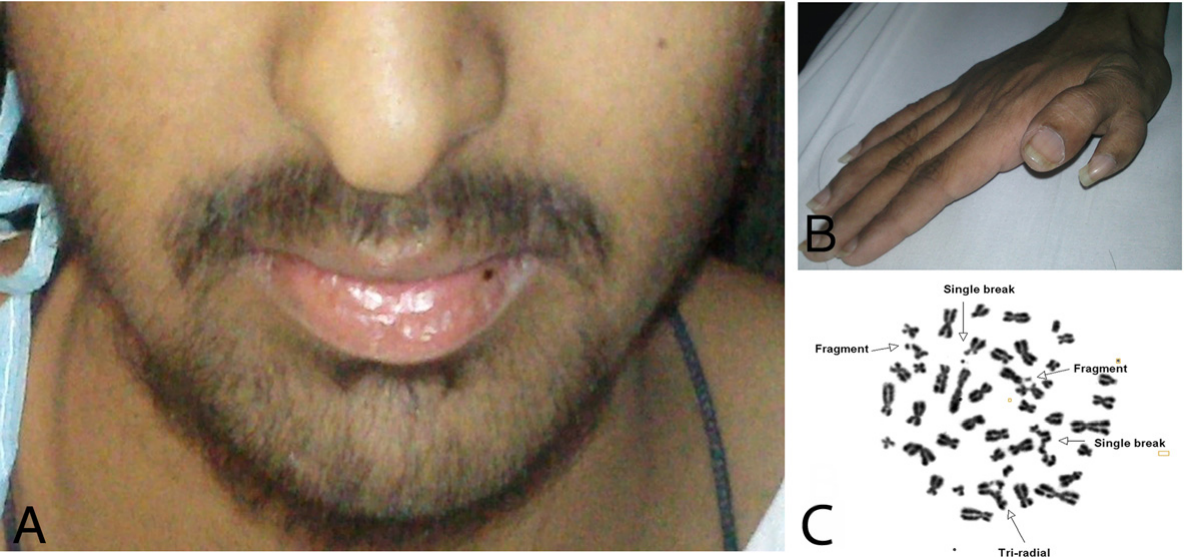
**Clinical and diagnostic features of the Fanconi anemia patient. (A)** Fanconi facies with tapering jaw. **(B)** Polydactyly. **(C)** Chromosomal breaks in peripheral blood.

**Figure 2:**
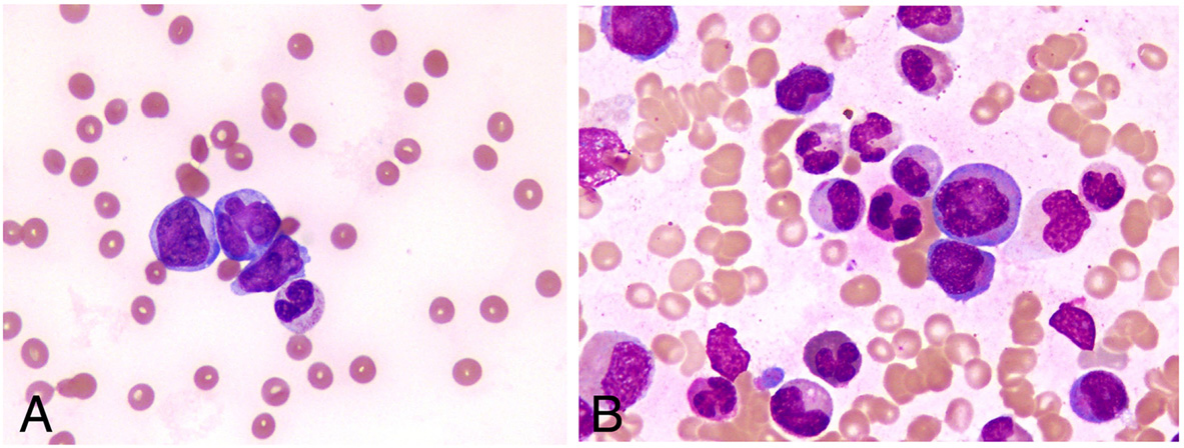
**Peripheral blood smear and bone marrow aspirate. (A)** Peripheral blood smear showing blast cells. **(B)** Bone marrow smear revealing a blast cell exhibiting an Auer rod.

**Figure 3:**
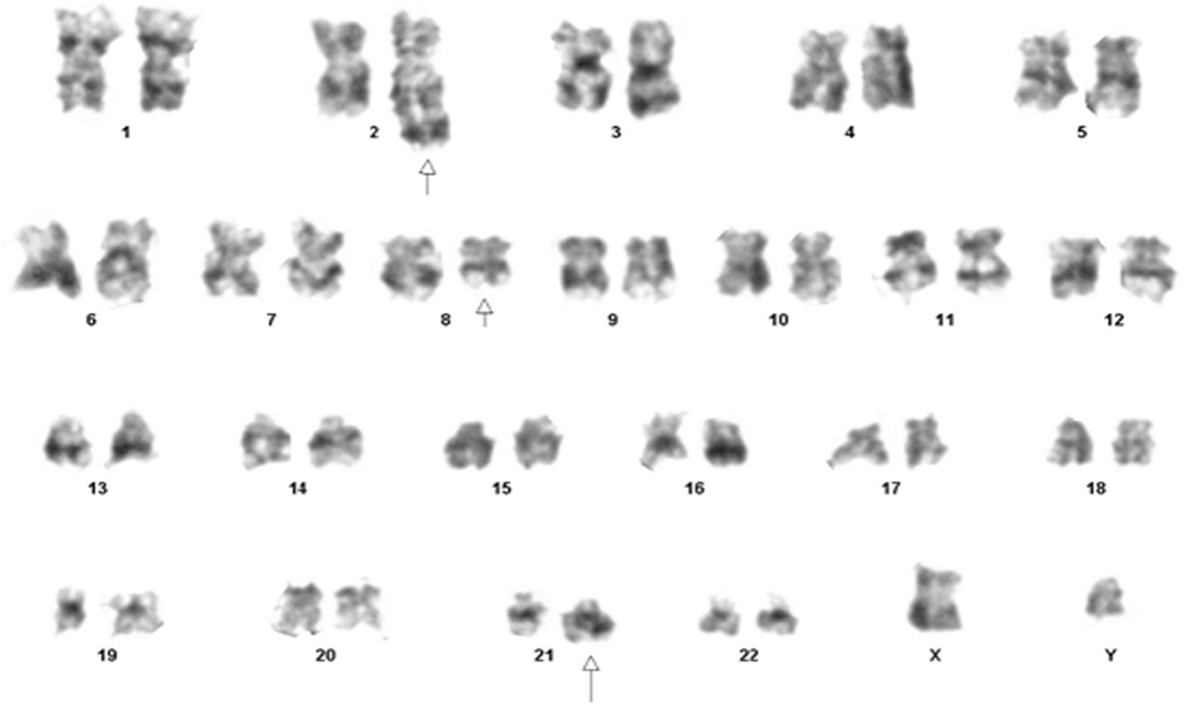
Bone marrow cytogenetics; Karyogram reveals translocation (8;21) and addition on chromosome 2 (arrow).

A 17 year old male presented to the emergency with a history of high grade fever since two weeks. Examination revealed short stature, tapering jaw (Figure 1A), pallor and thumb polydactyly (Figure 1B). There was no visceromegaly or lymphadenopathy. Previous complete blood counts were not available. Family history was insignificant. Complete blood count showed haemoglobin 3.4 gm/dl, MCV 100 fl and MCH 36 pg, white blood cell count 55.9 × 10 E9/L and platelet 8 × 10E9/L. Peripheral blood smear revealed 26% blast cells (Figure 2A). Bone marrow was hypercellular exhibiting infiltration with 21% blast cells comprising of myeloblasts and monoblasts with presence of auer rods (Figure 2B). Bone trephine was hypercellular showing diffuse infiltration with sheets of blast cells. The overall findings were consistent with acute myelomonocytic leukemia (AML M-4, according to F.A.B classification). The blasts cells expressed CD 33, CD13, HLA-DR, CD 117, CD34 antigens and cytoplasmic myeloperoxidase on immunophenotyping. Bone marrow cytogenetics revealed 46, XY, t (8:21) (q22; q22) [11]/ 46, XY, add (2) (q37),t (8; 21) [4]/46 XY [5] (Figure 3). Mutation analysis for NPM 1 and FLT3 ITD was negative while FLT3 D835 was positive. Based on his physical findings, peripheral blood was sent for analysis of chromosomal breakage (Figure 1C). There were 1.98 average number of breaks/cell in patient’s cells while control sample had 0.05 average number of breaks/cell. Tri-radial and complex figures were also noted. These features were consistent with Fanconi anemia and the patient was presumed to be transformed into AML at presentation.

Considering that the patient was high risk AML, option of subsequent allogeneic bone marrow transplant was discussed at consolidation therapy. Complete human leukocyte antigen (HLA) typing of the patient was performed and peripheral blood cytogenetics of his siblings was advised. He received induction chemotherapy with daunorubicin and cytarabine (3 + 7). During the hospital course, he developed febrile neutropenia for which he was empirically treated with antibiotics. Blood and urine cultures did not reveal growth of organisms. The neutropenia resolved on Day 21 and the patient became afebrile. Day 14 bone marrow revealed clearance of blast cells while minimal residual disease showed 0.03% blast cells. He was discharged and was advised follow up in outpatient clinic. However the patient was lost to follow up.

## Discussion

We have described a case of FA AML (AML that is presumed to be transformed from FA) with rare cytogenetics and poor prognosis due to positivity of FLT3 D835 mutation. AML is 500 fold more likely to develop in FA patients than the general population [9] and approximately one third of the patients with FA initially present with AML [8]. It has been reported that approximately 0.18% of newly diagnosed AML may have been transformed from FA [10]. This emphasizes the importance of identifying those patients with FA who are more likely to transform into AML and bone marrow cytogenetics may be the effective tool for this purpose. If specific cytogenetic abnormalities known to be associated with transformation to AML are known, the individual risk of developing leukemia can be predicted.

The most frequent chromosomal abnormalities in FA AML were gain of 1q, monosomy 7, and gain of 3q [7,11,12] while t(8;21), t(9;11), t(6;9), inv(16)(p13q22) and trisomy 8 were sole findings in de novo AML [11]. Other abnormalities that are common in the FA AML group include deletion 7q, gain of 13q, and deletion 20q [11]. P.A. Mehta et al. [12]. evaluated routine fluorescent in situ hybridization (FISH) analysis for 1q, 3q, monosomy 7 and 7q deletions on bone marrow samples from FA and they recommended that patients with small clones (<10%) should have regular follow up every 3 to 6 months with bone marrow FISH analysis while patients with large clones (>10%) and chromosome 7 abnormalities should undergo HSCT.

Our patient had none of the specific cytogenetic abnormalities known for FA AML except for t(8; 21) which is known to be more frequent in de novo AML and addition 2q, the significance of which is unknown. This indicates that AML should be considered as a transformation from FA even in the absence of known cytogenetic abnormalities for FA AML. This is important since FA cells are highly sensitive to chemotherapeutic agents leading to a poor outcome and the need for an early HSCT before transformation to leukemia occurs [7].

The rate of absolute neutrophil count (ANC) recovery after chemotherapy is known to be associated with certain cytogenetic abnormalities [10], such as t(8;21) which results in rapid ANC recovery (within 28 days post induction chemotherapy), while t(9;11) and t(6;9) (common in FA AML patients) lead to a delayed ANC recovery (>60 days) [11]. Our patient had t(8; 21) and he had a rapid ANC recovery within 21 days. Even though he had a good cytogenetic marker t(8; 21) for prognosis, the presence of FLT3 mutation placed him in poor prognostic group.

Literature review has revealed that there have not been any prospective studies or case reports from our region and the prevalence of specific cytogenetic abnormalities in our population needs to be known. It remains unclear in this case whether the cytogenetic abnormalities in the leukemic cells were present in the bone marrow prior to the diagnosis of leukemia. It is possible that Fanconi Anemia predisposes to the evolution of leukemic clones. It also remains unclear whether routine cytogenetic testing of patients with FA would identify patients at increased risk of developing AML and who are in need of HSCT. Systematic long-term follow-up studies needs to be carried out in our population.

## Conclusion

This case emphasizes the importance of identifying patients with AML who have transformed from FA in the absence of specific cytogenetic abnormalities. The recognition of specific cytogenetic abnormalities present in FA known to predispose to AML is crucial for early HSCT before transformation to leukemia.

## Consent

Written informed consent was obtained from the patient’s father for publication of this case report and any accompanying images. A copy of the written consent is available for review by the Editor-in-Chief of this journal. This case has also been exempted for ethical approval by the ethical review committee of Aga Khan University and Hospital (2561-Pat-ERC-13).

## Abbreviations

FA: Fanconi’s anemia; AML: Acute myeloid leukemia; FA AML: Acute myeloid leukemia transformed from Fanconi anemia; ANC: Absolute neutrophil count; ITD: Internal tandem domain; FISH: Fluorescent in situ hybridization; HLA: Human leukocyte antigen; MDS: Myelodysplastic syndrome; ALL: Acute lymphoblastic leukemia; HSCT: Haematopoietic stem cell transplant.

## Competing interests

The authors declare that they have no competing interests.

## Authors’ contributions

SH collected the information and wrote the manuscript. SNA is the primary physician who reviewed and edited the manuscript. Both authors read and approved the final manuscript.

## Authors’ information

SH is a fourth year resident in haematology department at the Aga Khan University and Hospital. SNA is an Associate professor and consultant haematologist at The Aga Khan University and Hospital.

## References

[B1] Rosenberg PS Greene MH Alter BP Cancer incidence in persons with Fanconi anemia Blood 2003 101 3 822 826 10.1182/blood-2002-05-1498 12393424

[B2] Shimamura A Alter BP Pathophysiology and management of inherited bone marrow failure syndromes Blood Rev 2010 24 3 101 122 10.1016/j.blre.2010.03.002 20417588 PMC3733544

[B3] Swift M Fanconi's anaemia in the genetics of neoplasia Nature 1971 230 5293 370 373 10.1038/230370a0 4927726

[B4] Mushtaq N Wali R Fadoo Z Saleem AF Acute lymphoblastic leukemia in a child with Fanconi's anaemia J Coll Physicians Surg Pak 2012 22 7 458 460 22747869

[B5] Li X Le Beau MM Ciccone S Yang FC Freie B Chen S Yuan J Hong P Orazi A Haneline LS Ex vivo culture of Fancc−/− stem/progenitor cells predisposes cells to undergo apoptosis, and surviving stem/progenitor cells display cytogenetic abnormalities and an increased risk of malignancy Blood 2005 105 9 3465 3471 10.1182/blood-2004-06-2483 15644418 PMC1895016

[B6] Alter BP Fanconi's anemia and malignancies Am J Hematol 1996 53 2 99 110 10.1002/(SICI)1096-8652(199610)53:2<99::AID-AJH7>3.0.CO;2-Z 8892734

[B7] Butturini A Gale RP Verlander PC Adler-Brecher B Gillio AP Auerbach AD Hematologic abnormalities in Fanconi anemia: an International Fanconi Anemia Registry study Blood 1994 84 5 1650 1655 8068955

[B8] Alter BP Cancer in Fanconi anemia, 1927–2001 Cancer 2003 97 2 425 440 10.1002/cncr.11046 12518367

[B9] Alter BP Giri N Savage SA Peters JA Loud JT Leathwood L Carr AG Greene MH Rosenberg PS Malignancies and survival patterns in the National Cancer Institute inherited bone marrow failure syndromes cohort study Br J Haematol 2010 150 2 179 188 20507306 10.1111/j.1365-2141.2010.08212.x PMC3125983

[B10] Rochowski A Rosenberg PS Alonzo TA Gerbing RB Lange BJ Alter BP Estimation of the prevalence of Fanconi anemia among patients with de novo acute myelogenous leukemia who have poor recovery from chemotherapy Leuk Res 2012 36 1 29 31 10.1016/j.leukres.2011.09.009 21974856 PMC4008327

[B11] Rochowski A Olson SB Alonzo TA Gerbing RB Lange BJ Alter BP Patients with Fanconi anemia and AML have different cytogenetic clones than de novo cases of AML Pediatr Blood Cancer 2012 59 5 922 924 10.1002/pbc.24168 22517793 PMC3407278

[B12] Mehta PA Harris RE Davies SM Kim MO Mueller R Lampkin B Mo J Myers K Smolarek TA Numerical chromosomal changes and risk of development of myelodysplastic syndrome–acute myeloid leukemia in patients with Fanconi anemia Cancer Genet Cytogenet 2010 203 2 180 186 10.1016/j.cancergencyto.2010.07.127 21156231

